# Exploring the Interplay between the Warburg Effect and Glucolipotoxicity in Cancer Development: A Novel Perspective on Cancer Etiology

**DOI:** 10.34172/apb.2024.049

**Published:** 2024-06-22

**Authors:** Maher Monir Akl, Amr Ahmed

**Affiliations:** ^1^Department of Chemistry, Faculty of Science, Mansoura University, 35516, Mansoura, Egypt.; ^2^The Public Health Department, Riyadh First Health Cluster, Ministry of Health, Saudi Arabia.

**Keywords:** Metabolic paradox, Cancer etiology, Glycolipid metabolism, Tumor development, Oncogenic pathways

## Abstract

The Warburg effect, first observed by Otto Warburg in the 1920s, delineates a metabolic phenomenon in which cancer cells exhibit heightened glucose uptake and lactate production, even under normoxic conditions. This metabolic shift towards glycolysis, despite the presence of oxygen, fuels the energy demands of rapidly proliferating cancer cells. Dysregulated glucose metabolism, characterized by the overexpression of glucose transporters and the redirection of metabolic pathways towards glycolysis, lies at the crux of this metabolic reprogramming. Consequently, the accumulation of lactate as a byproduct contributes to the creation of an acidic tumor microenvironment, fostering tumor progression and metastasis. However, recent research, notably proposed by Maher Akl, introduces a novel perspective regarding the role of glycolipids in cancer metabolism. Akl’s glucolipotoxicity hypothesis posits that aberrant glycolipid metabolism, specifically the intracellular buildup of glycolipids, significantly influences tumor initiation and progression. This hypothesis underscores the disruptive impact of accumulated glycolipids on cellular homeostasis, thereby activating oncogenic pathways and promoting carcinogenesis. This perspective aims to synthesize the intricate mechanisms underlying both the Warburg effect and glucolipotoxicity, elucidating their collective contributions to tumor growth and malignancy. By comprehensively understanding these metabolic aberrations, novel avenues for therapeutic intervention targeting the fundamental drivers of cancer initiation and progression emerge, holding promise for more efficacious treatment strategies in the future.

## Introduction

 Cancer, an ailment characterized by aberrant cell proliferation and invasion, remains a formidable challenge in the medical domain. Comprehending the fundamental mechanisms propelling tumor genesis is imperative for devising efficacious therapeutic modalities.^1^ The phenomenon known as the Warburg effect, coined after Otto Warburg, a Nobel laureate who initially delineated the altered metabolic profile of cancer cells, has garnered substantial attention.^2^ This metabolic shift entails heightened glucose uptake and lactate production, notwithstanding the presence of oxygen, thereby favoring glycolysis over oxidative phosphorylation, a phenomenon long recognized as a hallmark of cancer.^3^ The enigmatic preference of cancer cells for a less efficient energy production route, despite the availability of oxygen, has instigated extensive inquiry into the molecular and cellular underpinnings of the Warburg effect and its ramifications for tumorigenesis.^4^ One plausible hypothesis to elucidate the Warburg effect is the transient closure of glucose transporters on cancer cell membranes, purportedly triggered by the accumulation of glycolipids, thereby perturbing cellular homeostasis. This transient closure facilitates metabolizing excess glucose before normalizing glucose uptake, prompting cancer cells to resort to anaerobic glycolysis, leading to lactate accumulation and the creation of an acidic tumor microenvironment.^5,6^ This acidic milieu not only fosters tumor progression but also engenders immune suppression, angiogenesis, and invasiveness.^7^ While Warburg primarily focused on metabolic alterations, Maher Akl proposed a more comprehensive viewpoint, positing that dysregulated glycolipid metabolism, particularly glycolipid accumulation within cells, drives tumor growth.^8^ This perspective expands upon Warburg’s elucidation and underscores the pivotal role of glucolipotoxicity in cancer pathogenesis. This Perspective discourse aims to delve into the mechanisms underpinning the Warburg effect and glycolipid metabolism dysregulation, offering insights into the fundamental drivers of cancer and potentially unveiling novel therapeutic targets. By elucidating these processes, this Perspective endeavors to augment the corpus of knowledge concerning tumorigenesis, furnishing a framework for further scientific inquiry in this realm.

## Methodology

 To elucidate the intricate mechanisms underlying both the Warburg effect and glucolipotoxicity in tumor development, a comprehensive review of existing literature was conducted. Primary databases including PubMed, Web of Science, and Scopus were systematically searched for relevant articles published up to January 2024. The search strategy employed a combination of keywords such as “Warburg effect,” “glucolipotoxicity,” “cancer metabolism,” “glycolipid metabolism,” and “tumor development.” Articles were screened based on their relevance to the topic and inclusion of mechanistic insights into the metabolic alterations observed in cancer cells. Studies focusing on the molecular pathways involved in glucose metabolism, glycolysis regulation, and the role of glycolipids in cancer progression were prioritized. Additionally, seminal works by Otto Warburg and contemporary perspectives proposed by Maher Akl were meticulously reviewed to establish a comprehensive understanding of the historical context and recent advancements in the field. Data synthesis involved categorizing findings according to the key themes identified, namely the Warburg effect and glucolipotoxicity, and their respective implications for tumor growth. Mechanistic pathways elucidated in the literature were critically analyzed to delineate the interplay between dysregulated glucose and glycolipid metabolism in cancer cells. Moreover, emphasis was placed on identifying potential points of convergence or divergence between the two phenomena, shedding light on their synergistic or antagonistic effects on tumorigenesis. Furthermore, the methodology involved in vitro and in vivo studies exploring the effects of glycolipid accumulation on glucose transporter dynamics and metabolic reprogramming was scrutinized. The role of advanced imaging techniques, metabolomics, and genetic manipulation in unraveling the complexities of cancer metabolism was also assessed to ascertain the reliability and validity of experimental findings. Overall, this methodology provided a robust framework for synthesizing diverse strands of evidence and generating novel insights into the multifaceted interplay between the Warburg effect and glucolipotoxicity in driving tumor development.

 By critically evaluating existing literature and integrating diverse perspectives, this study aims to contribute to the advancement of knowledge in cancer biol3ogy and pave the way for the identification of novel therapeutic targets.

## Glucolipotoxicity

 Glucolipotoxicity denotes the deleterious repercussions stemming from heightened levels of glycolipids within cellular compartments. This perturbation arises from an imbalance or dysregulation in lipid metabolism, particularly in glycolipid synthesis and degradation processes.^9^

**Figure 1:**
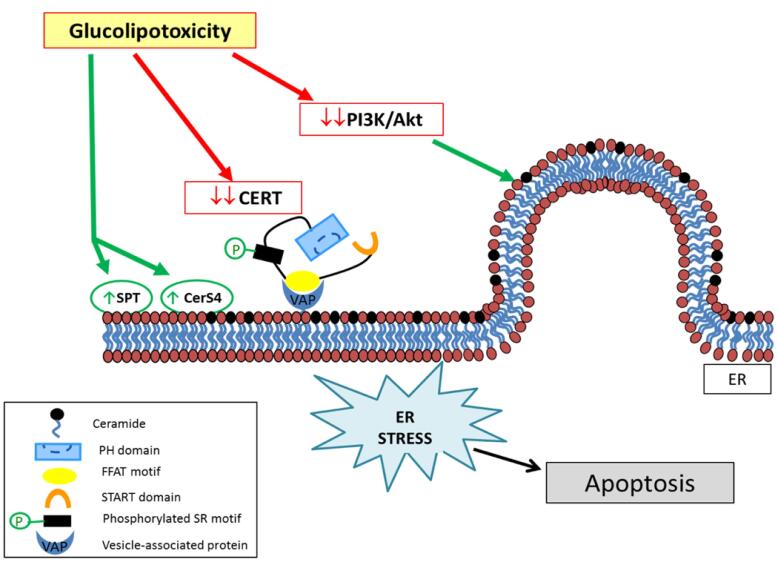
Classic pathways of endoplasmic reticulum (ER) stress and the unfolded protein response (UPR) are intricately linked to the pathophysiology of metabolic disorders such as lipotoxicity and glucotoxicity. The ER transmembrane sensors, including protein kinase RNA-like endoplasmic reticulum kinase (PERK), activating transcription factor 6 (ATF6), and IRE1, play pivotal roles in sensing misfolded proteins within the ER lumen. These sensors initiate a cascade of signaling events to restore ER homeostasis and alleviate cellular stress. For instance, ATF6 undergoes proteolytic cleavage in the Golgi apparatus upon ER stress, leading to its activation as a transcription factor. Similarly, IRE1 functions as both an endoribonuclease and a protein kinase, mediating the splicing of X-box binding protein 1 (XBP-1) mRNA and subsequent upregulation of ER stress response genes. PERK activation results in the phosphorylation of eukaryotic initiation factor 2 (eIF2)-α, thereby attenuating global protein translation while promoting the synthesis of select proteins, including ATF4, involved in adaptive UPR signaling. The adaptive UPR encompasses mechanisms such as the induction of protein chaperones and upregulation of ER-associated degradation (ERAD) to facilitate the clearance of misfolded proteins. However, sustained ER stress can overwhelm these adaptive responses, leading to apoptosis mediated by CCAAT/enhancer-binding protein homologous protein (CHOP) and other downstream effectors. Notably, in the context of metabolic diseases, ER stress-induced apoptosis contributes to pancreatic β-cell dysfunction, insulin resistance, and hepatic steatosis. Moreover, emerging evidence implicates ER stress in the pathogenesis of obesity-related inflammation and atherosclerosis, underscoring its broad relevance in metabolic dysregulation. The terminal UPR culminates in apoptotic signaling cascades, involving molecules such as caspase 12, TNF receptor-associated factor 2 (TRAF2), and c-Jun N-terminal kinase (JNK), which orchestrate cell death pathways in response to unresolved ER stress. Understanding the intricate crosstalk between ER stress and metabolic perturbations provides valuable insights into the pathogenesis of obesity, diabetes, and related disorders, highlighting potential therapeutic targets for intervention.^19^

 Glycolipids, comprising a carbohydrate moiety linked to a lipid tail, serve pivotal roles in diverse cellular functions.^10^ The genesis of glucolipotoxicity implicates various factors. Notably, excessive glucose influx into cells prompts an overproduction of glycolipids, surpassing the cellular machinery’s capacity for degradation, thereby fostering glycolipid accumulation.^11^ Moreover, insulin resistance, a hallmark of disorders like type 2 diabetes, exacerbates glucolipotoxicity.^12^ Impaired insulin responsiveness culminates in heightened glucose and fatty acid levels in the bloodstream, facilitating glycolipid synthesis and subsequent intracellular accumulation.^13,14^ The adverse effects of glucolipotoxicity emanate from the interference of accumulated glycolipids with essential cellular processes. Excessive glycolipid buildup disrupts lipid bilayers in cellular membranes, compromising their integrity and fluidity, thereby impeding the proper function of membrane-bound proteins and receptors, eliciting aberrant signaling and cellular responses.^15,16^ Furthermore, glucolipotoxicity induces endoplasmic reticulum (ER) stress, characterized by the accumulation of unfolded or misfolded proteins within the ER.^17^ Excessive glycolipids disrupt ER homeostasis, overwhelming the folding machinery and precipitating a stress response (Figure 1). Prolonged ER stress can activate inflammatory cascades and initiate apoptotic signaling, culminating in cellular dysfunction or demise.^18^

## Cellular repair mechanisms following glucolipotoxicity

**Figure 2:**
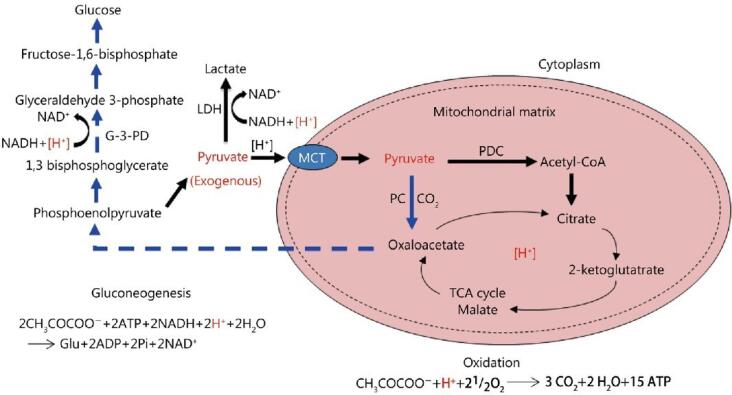
The metabolic fate of exogenous pyruvate and its impact on [H + ] consumption is elucidated through various pathways; Firstly, pyruvate undergoes oxidation into CO2 and H2O, a process that consumes an equimolar quantity of [H + ]. Secondly, the reduction of pyruvate to lactate also consumes an equimolar amount of [H + ]. Finally, the conversion of pyruvate into glucose via gluconeogenesis entails the consumption of a double-molar quantity of [H + ]. These processes highlight the dynamic interplay between pyruvate metabolism and [H + ] regulation within the cellular milieu. Furthermore, it’s essential to delineate the localization and transport mechanisms involved in these metabolic pathways. Monocarboxylate transporters (MCTs) facilitate the transport of lactate across cellular membranes. The pyruvate dehydrogenase complex (PDC) mediates the conversion of pyruvate to acetyl-CoA within mitochondria, while pyruvate carboxylase (PC) catalyzes pyruvate’s conversion to oxaloacetate, a precursor for gluconeogenesis. Additionally, the redox cofactors NAD + and NADH play pivotal roles in these metabolic reactions, undergoing oxidation and reduction, respectively. Lactic dehydrogenase (LDH) catalyzes the interconversion between pyruvate and lactate, while the tricarboxylic acid (TCA) cycle orchestrates the complete oxidation of acetyl-CoA to CO2 within mitochondria. These intricate metabolic pathways underscore the pivotal role of pyruvate in cellular energy metabolism and pH regulation, with [H + ] serving as a crucial determinant of cellular homeostasis^27^ The Creative Commons Attribution 4.0 International License (http://creativecommons.org/licenses/by/4.0/).

 Glucolipotoxicity, stemming from elevated glycolipid levels within cells, instigates a cascade of cellular repair mechanisms aimed at ameliorating damage and reinstating cellular equilibrium. Central to this repair process is the shutdown of glucose transporters, coupled with the engagement of alternative metabolic pathways for glucose disposal in the absence of oxygen.^20,21^ This intricate orchestration safeguards cellular viability and forestalls further glycolipid-induced detriment. Upon encountering glucolipotoxic stress, cells invoke a defensive strategy by downregulating glucose transporters, notably GLUT4, on the cell membrane.^22^ This downregulation curtails glucose influx, thereby diminishing the substrate available for glycolipid synthesis. By impeding glucose entry, the cell endeavors to arrest further glycolipid accumulation, thus alleviating the toxic burden. Simultaneously, in oxygen-deprived conditions, cells pivot to anaerobic metabolic pathways for glucose disposal.^23^ Glycolysis, pivotal in this anaerobic sugar disposal mechanism, metabolizes glucose into pyruvate. However, under normoxic conditions, pyruvate undergoes mitochondrial oxidation.^24^ In hypoxic settings, pyruvate conversion to lactate via lactate dehydrogenase ensues. This pyruvate-lactate conversion serves dual purposes: it regenerates NAD + from NADH, vital for sustaining glycolysis, and facilitates glucose expulsion from the cell. Lactate egress, facilitated by monocarboxylate transporters (MCTs), prevents further glucose accumulation, alleviating glucolipotoxicity-induced stress. Nonetheless, this anaerobic recourse harbors repercussions. Extracellular lactate accumulation can induce acidification, perturbing cellular pH and potentially compromising cellular processes (Figure 2). Moreover, prolonged reliance on anaerobic metabolism may engender diminished ATP production, given the superior efficiency of oxidative phosphorylation in mitochondrial ATP generation.^25,26^

## Accumulation of lactic acid: cellular response to anaerobic glucose metabolism and the role of lactic acid in inhibiting apoptotic enzymes

**Figure 3:**
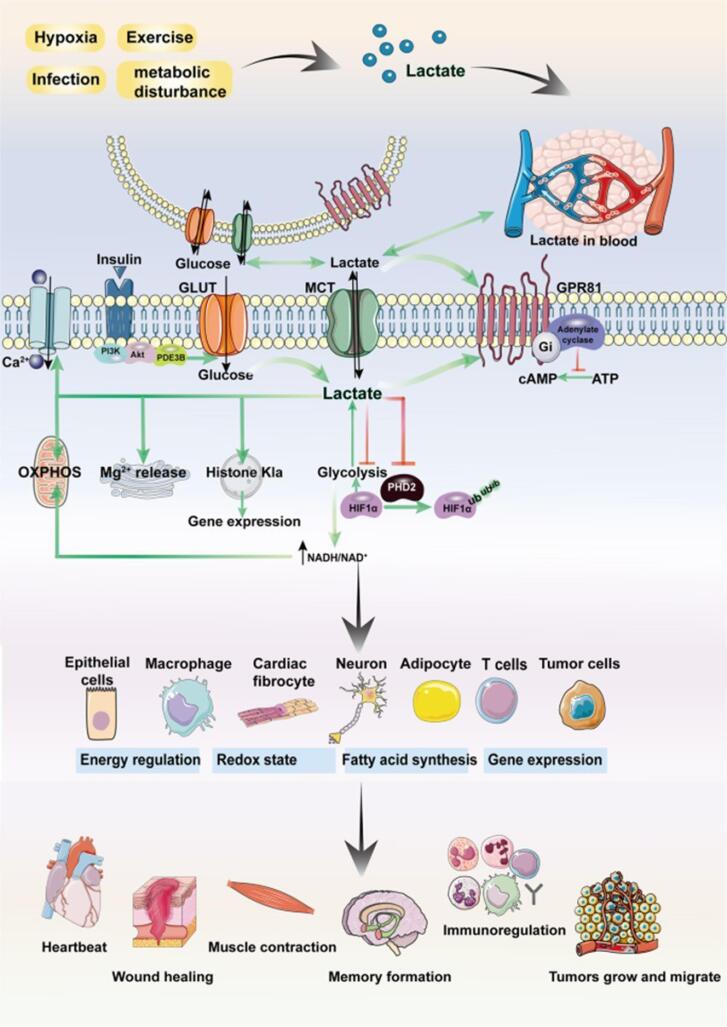
Lactate plays a multifaceted role in regulating cellular physiological and pathological processes, intricately linked to the overarching theme of the manuscript. Beyond its intracellular production, lactate can traverse cellular boundaries through intercellular shuttling mechanisms involving nonchannel pathways or monocarboxylate transporter 1 (MCT1). As both a signaling molecule and metabolic substrate, lactate participates in diverse cellular functions including glucose metabolism, fatty acid synthesis, maintenance of redox homeostasis, and post-translational modification (PTM) of proteins. Moreover, as a ligand for G protein-coupled receptor 81 (GPR81), lactate activates the GPR81 signaling cascade. Research has demonstrated lactate's involvement in regulating critical physiological processes such as muscle contraction, wound healing, memory formation, and tumor development. These diverse roles underscore lactate's significance in orchestrating cellular responses and maintaining tissue homeostasis.^36^ The Creative Commons Attribution 4.0 International License (http://creativecommons.org/licenses/by/4.0/).

 In the absence of oxygen, cells resort to anaerobic metabolism to generate energy from glucose. This metabolic adaptation leads to the accumulation of lactic acid as a byproduct of anaerobic glycolysis. The build-up of lactic acid plays a crucial role in cellular response and survival by inhibiting specific enzymes responsible for programmed cell death, known as apoptosis.^28^ Anaerobic glycolysis metabolism initiates with glycolysis, a process that breaks down glucose into pyruvate. In the presence of oxygen, pyruvate enters the mitochondria for further oxidation through the tricarboxylic acid (TCA) cycle and oxidative phosphorylation. However, under anaerobic conditions, pyruvate is converted into lactic acid through the enzymatic action of lactate dehydrogenase (LDH).^29^ The accumulation of lactic acid serves two critical functions: metabolic and cytoprotective. Metabolically, the conversion of pyruvate to lactic acid allows for the regeneration of NAD + from NADH, ensuring the continuous operation of glycolysis, which is dependent on NAD + availability. This metabolic function helps sustain cellular energy production in the absence of oxygen.^30^ Cytoprotective, lactic acid inhibits apoptotic enzymes, preventing programmed cell death. One enzyme affected by lactic acid is caspase-3, a key effector caspase involved in the execution phase of apoptosis. Lactic acid-induced acidification of the cytosol inhibits the activation of caspase-3, thereby preventing its proteolytic activity and the subsequent cleavage of cellular substrates required for apoptosis.^31,32^ Moreover, lactic acid accumulation can alter the pH balance within the cell. The increased acidity inhibits other enzymes involved in apoptosis, such as caspase-9 and caspase-8, by disrupting their conformation and activity. These enzymes play crucial roles in initiating the apoptotic cascade, and their inhibition by lactic acid contributes to the cell’s survival in oxygen-deprived conditions (Figure 3). While lactic acid accumulation serves as an adaptive response to anaerobic glucose metabolism, prolonged acidification can have detrimental effects on cellular function.^33,34^ Acidosis can disrupt protein structure, impair enzyme activity, and interfere with various cellular processes. Additionally, the reliance on anaerobic metabolism and lactic acid production for extended periods can result in reduced energy production and compromised cellular viability.^35^

## Glucolipotoxicity: Unraveling the link between metabolic disturbances and immune dysfunction

**Figure 4:**
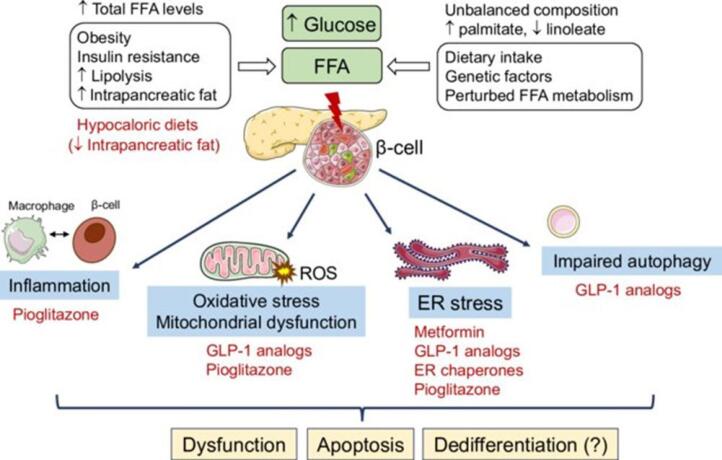
The molecular intricacies underlying the transition of cells towards a cancerous state under the influence of lipotoxicity and glucolipotoxicity, alongside potential therapeutic strategies, represent a pivotal realm of biological inquiry. Prolonged exposure to elevated levels of free fatty acids (FFAs), either in isolation or in conjunction with high glucose levels, triggers a cascade of stress responses within cells. These responses encompass diverse phenomena, including endoplasmic reticulum (ER) stress, heightened oxidative stress characterized by an overproduction of reactive oxygen species (ROS), mitochondrial dysfunction, inflammatory pathways activation, and compromised autophagic flux. The intricate crosstalk between these pathways may potentiate feed-forward mechanisms, exacerbating the detrimental effects of glucolipotoxic stress on cellular homeostasis. Ultimately, this complex interplay leads to cellular dysfunction, apoptosis, and potentially the acquisition of a malignant phenotype. Understanding the molecular intricacies governing the transition of cells towards malignancy in response to lipotoxic and glucolipotoxic conditions is imperative for developing targeted therapeutic interventions. Such strategies may involve interventions aimed at mitigating ER stress, reducing oxidative stress, restoring mitochondrial function, suppressing inflammation, and promoting efficient autophagic flux. By elucidating these mechanisms, novel therapeutic avenues may emerge, offering promising prospects for combating cancer and related malignancies.^38^ The CC BY-NC-ND license (https://creativecommons.org/licenses/by-nc-nd/4.0/).

 Emerging evidence suggests a link between glucolipotoxicity and immune dysfunction. Our research findings reveal that elevated levels of glucose and fatty acids not only trigger detrimental effects on cellular function but also have implications for immune responses. Specifically, we have observed that the increased glucose and fatty acids induce DNA damage, caspase-dependent apoptosis, and mitochondrial respiratory dysfunction. These cellular alterations are attributed to the concept of glucolipotoxicity, which arises from enhanced production of reactive oxygen species (ROS) and subsequent oxidative stress. It has been proposed that this oxidative stress disrupts the normal functioning of mitochondria, affecting their membrane potential and bioenergetics. In addition to the impact on cellular processes, our investigations have unveiled modifications in cell signaling pathways that are crucial for immune regulation. The Nrf-2/NFk-B/AMPK/mTOR-dependent signaling cascade, known to play a role in immune responses, was found to be altered when cells were exposed to high glucose and palmitic acid. Moreover, a dysregulated inflammatory response characterized by elevated levels of IL6 and PGE2 was observed in these conditions(Figure 4). These findings indicate that glucolipotoxicity exerts a multifaceted influence, not only on cellular function but also on immune signaling and inflammatory processes.^37^

## The paradoxical conditions that activate tumor growth: Unraveling the Warburg effect and elucidating the mechanisms

**Figure 5:**
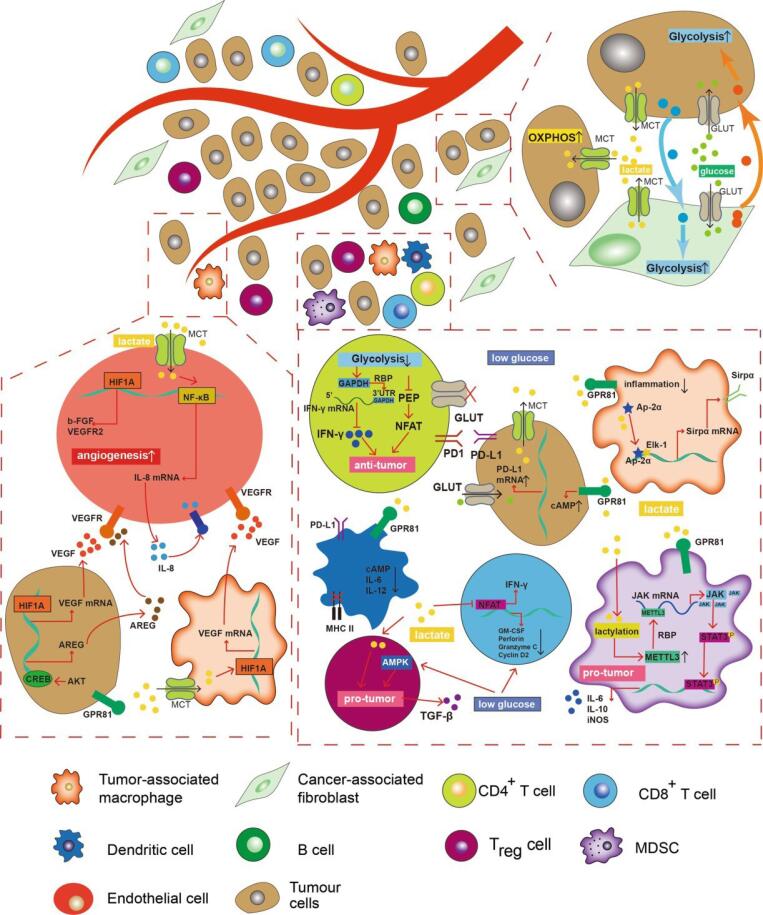
Glycolytic metabolism orchestrates a significant remodeling of the tumor microenvironment. Lactate, a byproduct of glycolysis, acts as a pivotal mediator, stimulating tumor cells and tumor-associated macrophages (TAMs) to secrete an array of factors that foster angiogenesis. Moreover, endothelial cells exhibit a responsiveness to extracellular lactate levels, promoting their proliferation. Conversely, glucose deprivation and extracellular acidosis exert profound suppressive effects on the anti-tumor activities of macrophages, CD4 + T cells, CD8 + T cells, and dendritic cells (DCs), while minimally impacting immunosuppressive cell populations such as myeloid-derived suppressor cells (MDSCs) and regulatory T cells (Tregs). Carcinoma-associated fibroblasts (CAFs) and cancer cells engage in a reciprocal enhancement of their glycolytic profiles. Additionally, a subset of tumor cells adopts a unique metabolic phenotype by assimilating lactate and undergoing oxidative metabolism, commonly referred to as “the reverse Warburg effect.” The directional arrows denote positive modulations or transitions, whereas blunt ends signify negative regulatory effects.^44^ The Creative Commons Attribution 4.0 International License (http://creativecommons.org/licenses/by/4.0/).

 The phenomenon commonly referred to as the Warburg effect, named after the distinguished scientist Otto Warburg, delineates the distinctive metabolic behavior of cancer cells, marked by heightened glucose uptake and lactate production, notwithstanding the presence of oxygen.^39^ This metabolic reprogramming, favoring glycolysis over oxidative phosphorylation, has garnered extensive scrutiny and is now acknowledged as a hallmark of cancer.^40^ A comprehensive comprehension of the seemingly paradoxical circumstances that fuel tumor proliferation via the Warburg effect is imperative for the advancement of innovative strategies targeting the fundamental mechanisms of cancer.^41^ This paper introduces the Maher Akl effect as an additional framework to elucidate the primary drivers of oncogenesis. The Warburg effect manifests through a complex interplay of mechanisms. Initially, cancer cells elevate the expression of glucose transporters, notably GLUT1 and GLUT3, on their cell membranes, augmenting glucose uptake and facilitating rapid proliferation, a process further potentiated by dysregulated signaling pathways such as the PI3K/Akt/mTOR pathway.^42^ Furthermore, cancer cells undergo metabolic reprogramming to prioritize glycolysis even under normoxic conditions, involving the upregulation of glycolytic enzymes like hexokinase II and pyruvate kinase M2 isoform, thus diverting glucose towards lactate production instead of entering the mitochondrial TCA cycle.^43^ The repercussions of the Warburg effect transcend altered glucose metabolism, encompassing the accumulation of metabolic intermediates such as lactate and certain amino acids, fostering an acidic tumor microenvironment that confers a selective advantage to tumor cells by dampening immune responses, promoting angiogenesis, and facilitating invasive behavior.^7^ In contrast, the Maher Akl effect introduces a novel perspective on the etiology of cancer, underscoring the significance of glucolipotoxicity in tumor initiation and progression. Maher Akl posits that the dysregulation of glucolipid metabolism, particularly the intracellular accumulation of glycolipids, serves as a key driver of tumorigenesis by disrupting essential cellular processes and fostering oncogenic pathways, akin to the Warburg effect (Figure 5).

## Discussion

###  Proposed primary cause of cancer according to the earlier described mechanisms

 The glucolipotoxicity hypothesis, as advanced by Maher Akl, introduces a distinctive viewpoint regarding the fundamental causality of cancer, diverging from the well-documented Warburg effect. While the Warburg effect scrutinizes the alterations in glucose metabolism and lactate production, the glucolipotoxicity hypothesis delves into the disruption of glucolipid metabolism and its implications in tumor initiation and progression. In this discussion, we delve into the mechanisms underpinning glucolipotoxicity, its ramifications on cellular functionality, and its potential role in driving tumor advancement, while juxtaposing the perspectives of Otto Warburg and Maher Akl.

 Glucolipotoxicity ensues when an excessive accumulation of glycolipids disrupts cellular equilibrium, impeding crucial cellular processes. A pivotal repercussion of this accumulation is the transient closure of glucose transporters, stymying further glucose uptake until the excess glucose can be metabolized. Subsequently, cells resort to anaerobic glycolysis, metabolizing glucose in the absence of oxygen and engendering an acidic milieu due to lactic acid accumulation. The resultant acidity within the tumor microenvironment fosters tumor growth by hindering apoptosis, the programmed cell death mechanism. Elevated acidity impedes enzymes pivotal for apoptosis initiation, such as caspase-3, caspase-9, and caspase-8, ultimately facilitating cell survival and tumor progression. Furthermore, in oxygen-deprived conditions, cells may adopt alternative metabolic pathways to sustain energy demands, including anaerobic glycolysis, conferring a selective advantage to cells harboring genetic aberrations conducive to tumorigenesis.

 Otto Warburg extensively elucidated this metabolic adaptation in his exploration of the Warburg effect, underscoring the significance of altered glucose metabolism in driving cancer cell proliferation. Nevertheless, Maher Akl’s glucolipotoxicity hypothesis transcends the metabolic perturbations elucidated by Warburg. Akl posits that the accumulation of glycolipids, stemming from dysregulated glucolipid metabolism, serves as the primary catalyst for tumor growth. The perturbation of cellular processes by accumulated glycolipids induces cellular dysfunction and activates oncogenic pathways, ultimately fostering tumor development.

 In comparing the viewpoints of Warburg and Akl, it becomes apparent that both underscore the pivotal role of metabolic dysregulation in cancer genesis. While Warburg accentuates the metabolic shift to glycolysis and its sequelae, Akl emphasizes the dysregulation of glucolipid metabolism and its repercussions on cellular functionality. These disparate perspectives contribute to a comprehensive comprehension of the primary instigators of cancer and hold promise in guiding the formulation of innovative therapeutic modalities.

## Conclusion

 In conclusion, the proposed primary cause of cancer according to the glucolipotoxicity hypothesis involves the dysregulation of glycolipid metabolism and its implications for cellular function. Accumulated glycolipids disrupt cellular processes, promoting cell survival and tumor progression. Comparing the perspectives of Otto Warburg and Maher Akl allows for a more comprehensive understanding of the metabolic alterations in cancer and opens avenues for further research and therapeutic interventions.

## Competing Interests

 The authors declare that there are no conflicts of interest.

## Ethical Approval

 Not applicable.
